# Knowledge, attitudes, and adherence relating to COVID-19 and its prevention measures in high-risk districts of Uganda in 2020

**DOI:** 10.3389/fepid.2023.1068097

**Published:** 2023-05-02

**Authors:** Tubihemukama Methodius, Angella Musewa, Bernadette Basuta Mirembe, Doreen Birungi, Sarah Nitumusiima, Irene Naigaga, John David Kabasa, William Bazeyo

**Affiliations:** ^1^ College of Veterinary Medicine, Animal Resources and Biosecurity, Makerere University, Kampala, Uganda; ^2^ Africa One Health University Network, Kampala, Uganda; ^3^ Ministry of Health Kampala, Kampala, Uganda

**Keywords:** attitude, adherence, COVID-19, prevention, measures, knowledge

## Abstract

**Introduction:**

COVID-19 is an infectious disease caused by severe acute respiratory syndrome coronavirus 2. There were no licensed vaccines or explicit medicines available for treatment at the time of conducting this study. Public health and social measures (PHSM) have been widely adopted to reduce the transmission of COVID-19. Hence, assessing people's knowledge, attitudes, and adherence/practices related to the management of COVID-19 is crucial for identifying the factors that may promote or hinder adherence to the implementation of PHSM.

**Methods:**

We conducted a cross-sectional study in the Amuru, Kyotera, Wakiso, and Kampala districts of Uganda. We used a simple random sampling technique to select households and conducted face-to-face interviews in selected households. We administered questionnaires to respondents to assess the factors that promote or hinder adherence to and knowledge about PHSM implementation. We used a Likert scale to assess respondents’ attitudes toward COVID-19.

**Results:**

Out of the 270 respondents, 54 (20%), 73 (27%), 42 (15.6%), and 101 (37.4%) were from the Kampala, Amuru, Wakiso, and Kyotera districts, respectively. Most of the respondents had adequate knowledge (72.2%), a high level of adherence (63.7%), and approximately 57.8% had good attitudes relating to COVID-19 and its prevention measures. An inferential analysis revealed that people from the Kampala district had higher chances (odds ratio = 4.668) of having a high level of knowledge compared to people from the Amuru district. It was also found that respondents who had a high level of (adequate) knowledge were twice as likely to have good attitudes compared to those with a low level of knowledge. In addition, people with good attitudes were 2.5 times as likely to adhere to the COVID-19 prevention measures compared to those with poor attitudes.

**Conclusion:**

Most respondents had limited knowledge though the majority of them had adopted practices to prevent the spread of COVID-19. Respondents with low knowledge of COVID-19 need to be targeted, to improve their attitude toward the disease and their adherence to safe prevention practices.

## Introduction

COVID-19 is a contagious disease caused by severe acute respiratory syndrome coronavirus 2 (SARS-CoV-2) (1). The virus was first detected in Wuhan city, central China, in December 2019 (2). Cases were detected in other countries among international travelers who traveled from Wuhan to various countries (3). By February 2023, more than 6.8 million deaths worldwide and 3,628 cumulative deaths in Uganda had been recorded (4, 5). Currently, there are approved vaccines but no explicit medicines available for the treatment of COVID-19, and appropriate public health and social measures (PHSM) are key in preventing its transmission.

According to the WHO, PHSM are actions performed by individuals, institutions, communities, local and national governments, and international bodies to slow or stop the spread of COVID-19 (6). These measures can be individual and public, such as detecting and isolating cases, contact tracing and quarantine, and social and physical distancing (7). For PHSM to slow or stop the transmission of COVID-19, they must be implemented with the full engagement of the public. The extent to which the public adheres to the guidelines issued by the government is key.

COVID-19 is transmitted predominantly through respiratory droplets and, to a lesser extent, through contact with contaminated surfaces (8). A study conducted by Jones et al. highly indicated evidence of environmental contamination by patients with SARS-CoV-2 through respiratory droplets and fecal shedding, suggesting the environment is a potential medium of transmission; this supports the need for strict adherence to hand hygiene and other safety measures (9).

Uganda experienced the COVID-19 outbreak, with nearly 40,000 cases as of February 2021 (10). The government of Uganda imposed a total lockdown on most businesses and movements, both nationally and internationally, for easy containment of the disease, as was done in many other countries (11). The Ugandan Ministry of Health also invested heavily in community sensitization through media houses as well as mass testing, the equipping of healthcare units, and the recruitment of staff to increase the country's task force to fight the disease (12). By June 2020, it had been just 4 months since the confirmation of the first case in Uganda, and new cases were accumulating, with over four deaths, 1,154 accumulative cases, and over 1,000 recoveries (10). Community transmission was reported in various districts at the time of this study, given the intensive sensitization by the government. The Kampala and Wakiso districts recorded the highest number of community transmission cases whereas the Kyotera and Amuru districts were border districts at risk of continuous importation of the infection. The transmission of COVID-19 could be reduced by adhering to the standard operating procedure (SOP) (13). Therefore, the present study aimed to assess the knowledge, attitudes, and adherence among communities in high-risk districts and determine the possible drivers affecting adherence to PHSM for COVID-19. This study will add to the existing knowledge about the prevention and control of the spread of infectious diseases in low-income countries such as Uganda. The study helps to understand the different knowledge, practices, and attitudes about the PHSM regarding the border districts.

## Methods

### Study design and area

**Figure 1:**
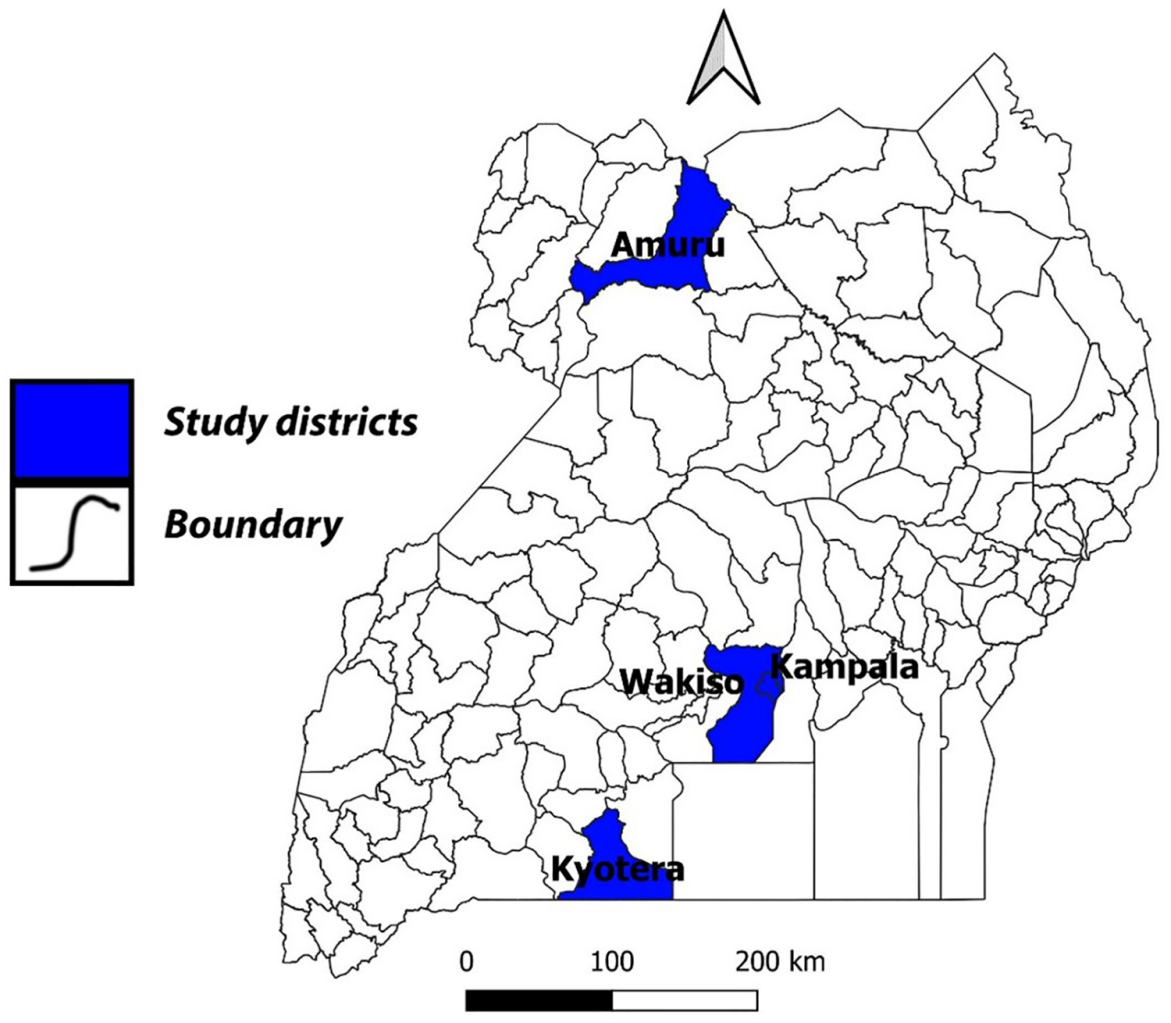
Map of Uganda showing the Amuru, Kyotera, Wakiso, and Kampala districts.

We conducted a cross-sectional study involving four districts: Amuru, Kyotera, Wakiso, and Kampala. These districts were selected because they registered the highest community and local transmission in Uganda at the time of writing the concept in June 2020. This was characterized by increased daily new cases within such communities, whose levels of knowledge, attitudes, and adherence to the prevention measures were unknown at the time. The Amuru district, in northern Uganda, hosts the Elegu border point, which is the major cross-border point to South Sudan. The Kyotera district, in southern Uganda, hosts two major cross-border points to Tanzania: Mutukula and Kasesero. The Kampala district, in the central region and home to the capital city of Uganda, hosts most national institutions. The Wakiso district, in the central region, surrounds Kampala and hosts the only international airport in Uganda, Entebbe. These areas have been illustrated on a map in Figure 1.

### Study population and sampling strategy

The present study was conducted among individuals in households within selected high-risk districts with community transmission. Emphasis was made to ensure that the head of the household responded to the questions since they were assumed to have sufficient knowledge about those living in the same household. We used households as our study unit to ensure different respondents were interviewed. The total number of people (population) in all the study districts was approximately 5,072,800 (https://www.citypopulation.de/en/uganda/admin/). Using the Taro Yamane formula, a total of 280 households was estimated to be selected from the four districts of study. We used a simple random sampling method to select households. Using information from the district health officers (DHOs) and village health teams (VHTs), high-risk sub-counties (with a high likelihood of human concentration, such as trading centers) were considered for the selection of households.

### Data collection

We conducted face-to-face interviews guided by a structured questionnaire administered to respondents of these selected households during the month of November 2020, when lockdown restrictions were partly loosened to all movement of extra public health workers. We collected data on demographic characteristics, including sex, age, level of education, exposure factors, and sources of information on COVID-19. Knowledge of COVID-19 was assessed with different questions and each plausible answer considered to be correct scored 1 point; incorrect responses scored 0 points. We assessed the attitudes of respondents using a 5-point Likert scale with questions modified appropriately for COVID-19. The responses were as follows: strongly disagree, disagree, neutral, agree, and strongly agree, with each scoring 1–5, respectively. We categorized responses using a 4-point Likert scale for practices toward COVID-19; these were scored 1 to 4 as always, sometimes, rarely, and never, respectively.

### Data management and analysis

Data were entered into an Excel spreadsheet and cleaned for inconsistencies and missing values. The cleaned data were then imported into SPSS version 23 for statistical analysis. Numerical data were presented as means and standard deviations. Categorical data were presented as frequencies and proportions. The variables that were used to evaluate each of the components (knowledge, attitude, and adherence) were first averaged for each respondent (row-wise) to generate a continuous column of means for each component. For each component, a mean of the means was computed. In addition, for each component, values above the mean of the means were categorized as adequate/good/high and those below the mean of the means as inadequate/poor/low, for knowledge, attitude, and adherence, respectively. The association between knowledge, attitude, and adherence with predisposing factors was determined using a chi-square test, and associations with related *p*-values <0.05 were considered to be significant (bivariate analysis level). The significant factors were then combined to run logistic regression models (multivariate analysis level).

## Results

The present study aimed to assess the knowledge, attitude, and adherence relating to COVID-19 prevention measures. It was conducted in four districts in Uganda: Amuru (73/270, 27%), Kyotera (101/270, 37.4%), Wakiso (42/270, 14.6%), and Kampala (54/270, 20.0%). Most (165/270, 61.1%) of the respondents were women, whereas 105 (38.9%) were men. The mean age of the respondents was 39 ± 13 years, and most of the respondents (114/270, 42.2%) were aged 30–44 years. The majority of the respondents had attained a primary education level (34.8%), whereas fewer (15.2%) of them did not have any formal education. Their major source of COVID-19 information was the media, including radio, television, and social media platforms such as WhatsApp and Facebook. Friends as a source of information included relatives, peer members, and any other person involved in the conveying of information without involving any media means (Table 1).

**Table 1:** Demographic characteristics of the respondents.

Variable	Level	Frequency	Percent
Sex	Male	105	38.9
	Female	165	61.1
Age (years)	15–29	101	37.4
	30–44	114	42.2
	45–59	35	13
	>59	20	7.4
District	Amuru	73	27
	Kampala	54	20
	Kyotera	101	37.4
	Wakiso	42	15.6
Marital status	In a relationship	179	66.3
	Single	91	33.7
Level of education	None	41	15.2
	Primary	94	34.8
	Secondary	90	33.3
	Tertiary	45	16.7
Occupation	Student	37	13.7
	Farmer	82	30.4
	Health worker	27	10
	Business	124	45.9
Means of transport	Public	25	9.3
	Private	28	10.4
	Other	217	80.4
Source of information	Media	182	67.4
	Friends	88	32.6

### Knowledge about COVID-19 and prevention measures

The majority (72.2%) of the respondents had adequate knowledge of COVID-19 and its prevention measures. This could be attributed to efforts by the Ministry of Health in educating the public about prevention and control measures. People from the districts of Wakiso (100%), Kyotera (63.5%), Kampala (100%), and Amuru (78.1%) registered adequate knowledge about COVID-19 and its prevention measures, except for Amuru (42.5%) (Table 2).

**Table 2:** Distribution of knowledge of COVID-19 prevention measures according to demographic characteristics.

Variable	Level	Inadequate	Adequate	Total	*p*-value
District	Amuru	8 (11)	65 (89)	73	0.000
	Kampala	38 (70.4)	16 (29.6)	54	
	Kyotera	16 (15.8)	85 (84.2)	101	
	Wakiso	13 (31)	29 (69)	42	
Sex	Male	22 (21)	83 (79)	105	0.046
	Female	53 (32.1)	112 (67.9)	165	
Marital status	In a relationship	46 (25.7)	133 (74.3)	179	0.285
	Single	29 (31.9)	62 (68.1)	91	
Age (years)	15–29	23 (22.8)	78 (77.2)	101	0.321
	30–44	32 (28.1)	82 (71.9)	114	
	45–59	12 (34.3)	23 (65.7)	35	
	>59	8 (40)	12 (60)	20	
Occupation	Student	11 (29.7)	26 (70.3)	37	0.956
	Farmer	21 (25.6)	61 (74.4)	82	
	Health worker	8 (29.6)	19 (70.4)	27	
	Business	35 (28.2)	89 (71.8)	124	
Level of education	None	15 (36.6)	26 (63.4)	41	0.055
	Primary	28 (29.8)	66 (70.2)	94	
	Secondary	16 (17.8)	74 (82.2)	90	
	Tertiary	16 (35.6)	29 (64.4)	45	
Means of transport	Public	11 (44)	14 (56)	25	0.080
	Private	10 (35.7)	18 (64.3)	28	
	Other	54 (24.9)	163 (75.1)	217	
Source of information	Media	62 (34.1)	120 (65.9)	182	0.001
	Friends	13(14.8)	75(85.2)	88	

Values are given as n (%).

#### Association between knowledge and demographic characteristics

The majority (72.2%) of the respondents had an adequate knowledge of COVID-19 control measures. The knowledge was associated significantly with district (*p* = 0.00), sex (*p* = 0.046), and source of information (*p* = 0.001) (Table 2). It is worth observing that people from Kyotera, Amuru, and Wakiso had a higher knowledge of COVID-19 control measures than those from Amuru and Kyotera. In fact, no respondents were found to have inadequate knowledge in Wakiso and Kampala. It was also discovered that the means of transport commonly used by a person was significantly associated with his/her knowledge. The majority of the men (69.5%) and women (71.5%) had an above-average knowledge of COVID-19 prevention measures.

The dependent variable was the level of knowledge, whereas the independent variables were those that were significantly associated with the level of knowledge in a chi-square test (district of residence, sex, and source of information). The factors were considered as significantly related to knowledge if the corresponding *p*-value was <0.05 (critical). People in the Kampala district were associated with increased odds [adjusted odds ratio (AOR) = 4.668, *p* = 0.003] of having adequate knowledge compared to those from the Amuru district as presented in Table 4.

Men were twice as likely to have adequate knowledge compared to women, although this relationship was not significant.

### Attitudes toward COVID-19 prevention measures

Most (57.8%) of the respondents had a good attitude toward COVID-19 and its prevention measures. The majority (75.9%) of the people strongly agreed that COVID-19 was a dangerous infectious disease. Most (72.2%) of the people strongly agreed that COVID-19 was a dangerous infectious disease. The good attitudes toward COVID-19 and its prevention may also be explained by the fact that most of the respondents strongly agreed that they were at risk of contracting the disease (53.7%) and that the transmission can be prevented (54.1%) (Table 3).

**Table 3:** Attitudes of respondents toward COVID-19 prevention and control measures.

Variable	Strongly disagree	Disagree	Neutral	Agree	Strongly agree
COVID-19 is a dangerous disease	13 (4.8)	6 (2.2)	3 (1.1)	43 (15.9)	205 (75.9)
COVID-19 is a very infectious disease	8 (3)	7 (2.6)	8 (3)	52 (19.3)	195 (72.2)
I am at risk of contracting COVID-19	15 (5.6)	22 (8.1)	15 (5.6)	73 (27)	145 (53.7)
Transmission of COVID-19 can be prevented	11 (4.1)	7 (2.6)	10 (3.7)	96 (35.6)	146 (54.1)
I would alert authorities in case had signs/symptoms of COVID-19	11 (4.1)	8 (3)	14 (5.2)	96 (35.6)	141 (52.2)
I would report unexplained deaths in my community to local authorities/health center	7 (2.6)	11 (4.1)	21 (7.8)	99 (36.7)	132 (48.9)
Healthcare providers can handle COVID-19 outbreaks very well	16 (5.9)	12 (4.4)	22 (8.1)	78 (28.9)	142 (52.6)
I would freely interact with a discharged person with a history of COVID-19 infection	36 (13.3)	32 (11.9)	21 (7.8)	67 (24.8)	114 (42.2)
I would feel safe wearing a mask to prevent COVID-19	15 (5.6)	29 (10.7)	29 (10.7)	77 (28.5)	120 (44.4)
Masks should also be worn at home	39 (14.4)	52 (19.3)	30 (11.1)	49 (18.1)	100 (37.0)
Hand washing helps protect me from COVID-19	10 (3.7)	10 (3.7)	18 (6.7)	73 (27)	159 (58.9)
Using a sanitizer is better than washing hands with soap when preventing COVID-19	123 (45.6)	74 (27.4)	37 (13.7)	16 (5.9)	20 (7.4)
Physical distancing is a feasible prevention of COVID-19 in Uganda	11 (4.1)	13 (4.8)	19 (7)	79 (29.3)	148 (54.8)
Institutional quarantine helps prevent the transmission of COVID-19	12 (4.4)	14 (5.2)	16 (5.9)	83 (30.7)	145 (53.7)

Values are given as n (%).

**Table 4:** Logistic regression for analysis of factors relating to knowledge of the respondents about COVID-19 and the prevention measures.

Variable	*p*-value	AOR	95% CI for AOR	
			Lower	Upper
District *(Amuru)*				
Kampala	0.003	4.668	1.695	12.855
Kyotera	0.001	0.232	0.094	0.57
Wakiso	0.133	1.983	0.812	4.843
Sex *(Female)*				
Male	0.092	1.764	0.911	3.414
Source of info *(Friends)*				
Media	0.072	2.083	0.936	4.638

AOR, adjusted odds ratio; CI, confidence interval.

#### Association between attitudes and demographic characteristics

The majority of the respondents from Kyotera, Kampala, and Amuru had a good attitude toward COVID-19 prevention measures, whereas most of the respondents from Wakiso (28/42, 66.7%) had a poor attitude. The association between district and attitude levels was significant (*p* = 0.006). There was also a significant association between the knowledge of COVID-19 prevention measures and the practice of these measures (*p* = 0.005), as presented in Table 5.

**Table 5:** The association between attitudes and demographic characteristics of the respondents.

Variable	Level	Attitude level		Total	*p*-value
		Good attitude	Poor attitude		
Sex	Male	61 (58.1)	44 (41.9)	105	0.933
	Female	95 (57.6)	70 (42.4)	165	
Age (years)	15–29	58 (57.4)	43 (42.6)	101	0.777
	30–44	63 (55.3)	51 (44.7)	114	
	45–59	22 (62.9)	13 (37.1)	35	
	>59	13 (65.0)	7 (35.0)	20	
District	Amuru	44 (60.3)	29 (39.7)	73	0.006
	Kampala	35 (64.8)	19 (35.2)	54	
	Kyotera	63 (62.4)	38 (37.6)	101	
	Wakiso	14 (33.3)	28 (66.7)	42	
Marital status	In a relationship	105 (58.7)	74 (41.3)	179	0.681
	Single	51 (56.0)	40 (44.0)	91	
Occupation	Student	21 (56.8)	16 (43.2)	37	0.207
	Farmer	40 (48.8)	42 (51.2)	82	
	Health worker	18 (66.7)	9 (33.3)	27	
	Business	77 (62.1)	47 (37.9)	124	
Level of education	None	19 (46.3)	22 (53.7)	41	0.395
	Primary	54 (57.4)	40 (42.6)	94	
	Secondary	55 (61.1)	35 (38.9)	90	
	Tertiary	28 (62.2)	17 (37.8)	45	
Means of transport	Public	14 (56.0)	11 (44.0)	25	0.935
	Private	17 (60.7)	11 (39.3)	28	
	Other	125 (57.6)	92 (42.4)	217	
Knowledge	Inadequate	56 (70.9)	23 (29.1)	79	0.005
	Adequate	100 (52.4)	91 (47.6)	191	

At the multivariate level, logistic regression was conducted. Respondents with adequate knowledge were significantly associated with increased odds of having good attitudes compared to those with limited knowledge [*p* = 0.016, 95% confidence interval (CI) = 1.161–4.256] as presented in Table 7. People from Wakiso had increased odds of having a good attitude toward COVID-19 prevention measures compared to people from the Amuru district.

### Adherence to the recommended guidelines for the prevention and control of COVID-19

Most (63.7%) of the people adhered to the practices recommended by the Ministry of Health to prevent the spread of COVID-19. However, some guidelines were not followed, for example most of the people never washed their hands routinely (73.7%) as recommended by the Ministry of Health (Table 8). The majority of the people also never avoided masses of people (63.0%) as well as the cleaning of surfaces (45.2%). Similarly, most of the people avoided sharing masks (74.8%), given that most of them did not even wear masks (59.6%).

The adherence to COVID-19 prevention measures was associated with knowledge about these measures, the attitude of the people toward these measures, and the district (location). The associations were determined using chi-square tests, and the associations were rendered significant if the *p*-value was <0.05 (the critical value). There was a significant association between the district of the respondents and adherence to COVID-19 prevention measures.

High levels of adherence were observed among people aged 45–59 years compared to those who were aged 60 years or above. The majority of married people (65.4%) had a high level of adherence to the measures compared to unmarried people (60.4%) as presented in Table 9.

The determination of the factors influencing the practice of respondents about COVID-19 prevention measures involved logistic regression. The dependent variable was the practice level, whereas the independent variables were those that were significantly associated with the practice level in a chi-square test (district of residence, level of knowledge, and attitude). The factors were considered to be significantly related to knowledge if the corresponding *p*-value was <0.05 (critical value). The results were presented in Table 10. People in Wakiso were significantly associated with reduced odds of thoroughly practicing the prevention measures compared to those in the Amuru district (*p* < 0.01, AOR = 0.255, 95% CI = 0.085–0.762).

People with a good attitude were 2.5 times as likely to adhere to the COVID-19 prevention measures compared to those with a poor attitude. People with adequate knowledge were 28 times as likely to adhere to the COVID-19 prevention measures compared to those with poor attitudes.

## Discussion

During the year 2020, the world was hit by continuous strong waves of the COVID-19 pandemic (14). Uganda, like other low-income countries that are characterized by a weak and less financed health sector, was faced with difficulties in the prevention and containment of the disease. Many government agencies and ministries, such as the Ministry of Health, and stakeholders carried out campaigns to sensitize the public about SOPs aimed at increasing knowledge of the pandemic, as well as adherence to the procedures to reduce the spread and contain the pandemic. Therefore, the present study aimed to assess the knowledge, attitude, and adherence of people relating to these prevention measures, given the government of Uganda's and stakeholders’ sensitization. The results of this study could be used to evaluate the impact of government and stakeholder sensitization, and the methods used on people's knowledge and attitudes as well as their practice/adherence. This evaluation can also be used to identify the gaps and strengths in these campaigns for future improvement in case of similar outbreaks.

Mitigation strategies, including PHSM, have been employed in various countries to limit the transmission of COVID-19 and contain it. As highlighted by Wilder-Smith et al., the containment of the pandemic depends on the success of preventive measures that have been instituted (15). This has been evident in many countries, including Uganda, Tanzania, as well as China and Germany (16–18). In this study, most respondents had a good attitude toward COVID-19 prevention and control measures instituted by the government of Uganda. For instance, the majority of respondents reported having avoided crowded places, practiced social distancing, avoided sharing masks, and washed their hands with soap regularly; however, these may be subjective given the fact that they were individual reports. These findings were in agreement with those of Amodan et al. (19), who reported that people in Uganda observed physical distancing, as well as avoided touching their faces. Other studies also registered similar findings (20, 21). In this study, a significant number of respondents were aware that they were at risk of contracting COVID-19. Similarly, Kasozi et al. (22) and Okaka and Omondi (23) reported that respondents were aware of the risk of contracting COVID-19 but the implementation of practices such as hand washing was problematic and challenging in Uganda and Kenya, respectively. This could be combated by investing heavily in the sensitization of the public to induce behavior change in communities, especially in future pandemics and epidemic outbreaks, such as the common Ebola and Marburg outbreaks, since adequate knowledge is often associated with good attitudes and practices (24).

In this study, most of the respondents had adequate knowledge of COVID-19 and its prevention measures. This could be explained by the fact that most of the respondents were aware of the transmission routes as well as the signs and symptoms of COVID-19 (Table 6), given the government of Uganda's sensitization programs. Similar findings have been reported in Tanzania as well as Kenya in 2020, where more than 80% of the respondents had good knowledge of COVID-19 (25). The findings were also similar to those from a study conducted in Kenya in 2020 in which the authors revealed that most of the people had adequate knowledge of COVID-19 (26).

**Table 6:** Knowledge assessment of COVID-19 and prevention measures.

Variable	No	Yes
COVID-19 is transmitted through contact with an infected surface	118 (43.7)	152 (56.3)
COVID-19 is transmitted through air droplets from an infected person	91 (33.7)	179 (66.3)
COVID-19 is transmitted through engaging in masses	154 (57)	116 (43)
COVID-19 can be contracted through the nose	33 (12.2)	237 (87.8)
COVID-19 can be contracted through the mouth	47 (17.4)	223 (82.6)
COVID-19 can be contracted by touching the eyes	118 (43.7)	152 (56.3)
Coughing is a sign of COVID-19	56 (20.7)	214 (79.3)
Fever is a sign of COVID-19	89 (33)	181 (67)
Difficulty breathing is a sign of COVID-19	126 (46.7)	144 (53.3)
Sneezing is a sign of COVID-19	102 (37.8)	168 (62.2)
Washing hands prevents the spread of COVID-19	55 (20.4)	215 (79.6)
Wearing a mask prevents the spread of COVID-19	55 (20.4)	215 (79.6)
Physical distance prevents the spread of COVID-19	101 (37.4)	169 (62.6)
Disinfection prevents the spread of COVID-19	204 (75.6)	66 (24.4)
Touching of soft body parts may lead to the contraction of COVID-19	195 (72.2)	75 (27.8)
Masks should cover both the nose and mouth	21 (7.8)	249 (92.2)
I should wash my hands for more than 20 s	54 (20.0)	216 (80.0)

Values are given as n (%).

**Table 7:** Logistic regression results factors relating to attitude levels.

Variable (reference category)	*p*-value	AOR	95% CI for AOR	
Knowledge (*Inadequate)*			Lower	Upper
Adequate	0.016	2.223	1.161	4.256
District (*Amuru)*				
Kampala	0.124	0.528	0.234	1.192
Kyotera	0.428	0.77	0.404	1.469
Wakiso	0.135	1.945	0.812	4.657

AOR, adjusted odds ratio; CI, confidence interval.

**Table 8:** Adherence to the recommended guidelines for the prevention and control of COVID-19.

Variable	Always	Sometimes	Rarely	Never
Wears mask	2 (0.7)	28 (10.4)	79 (29.3)	161 (59.6)
Washes hands	3 (1.1)	17 (6.3)	51 (18.9)	199 (73.7)
Cleans surface	36 (13.3)	61 (22.6)	51 (18.9)	122 (45.2)
Does PD	7 (2.6)	32 (11.9)	73 (27)	158 (58.5)
Avoids masses	10 (3.7)	25 (9.3)	65 (24.1)	170 (63.0)
Proper respiratory etiquette	10 (3.7)	59 (21.9)	75 (27.8)	126 (46.7)
Avoids sharing mask	25 (9.3)	28 (10.4)	15 (5.6)	202 (74.8)
Respects curfew	14 (5.2)	22 (8.1)	52 (19.3)	182 (67.4)

Values are given as n (%).

PD, physical distancing.

**Table 9:** The association between demographic characteristics and adherence to COVID-19 prevention measures.

Variable	Category	Adherence level		Total	*p*-value
		Low	High		
Sex	Male	35 (33.3)	70 (66.7)	105	0.42
	Female	63 (38.2)	102 (61.8)	165	
Age (years)	15–29	38 (37.6)	63 (62.4)	101	0.05
	30–44	48 (42.1)	66 (57.9)	114	
	45–59	6 (17.1)	29 (82.9)	35	
	>59	6 (30)	14 (70)	20	
District	Amuru	24 (32.9)	49 (67.1)	73	0.00
	Kampala	8 (14.8)	46 (85.2)	54	
	Kyotera	53 (52.5)	48 (47.5)	101	
	Wakiso	13 (31)	29 (69)	42	
Marital status	In a relationship	62 (34.6)	117 (65.4)	179	0.43
	Single	36 (39.6)	55 (60.4)	91	
Occupation	Student	12 (32.4)	25 (67.6)	37	0.33
	Farmer	30 (36.6)	52 (63.4)	82	
	Health worker	6 (22.2)	21 (77.8)	27	
	Business	50 (40.3)	74 (59.7)	124	
Level of education	None	21 (51.2)	20 (48.8)	41	0.07
	Primary	37 (39.4)	57 (60.6)	94	
	Secondary	26 (28.9)	64 (71.1)	90	
	Tertiary	14 (31.1)	31 (68.9)	45	
Means of transport	Public	8 (32)	17 (68)	25	0.57
	Private	8 (28.6)	20 (71.4)	28	
	Other	82 (37.8)	135 (62.2)	217	
Attitudes	Good	62 (54.4)	52 (45.6)	156	0.00
	Poor	36 (23.1)	120 (76.9)	114	
Knowledge	Inadequate	16 (20.3)	63 (79.7)	79	0.00
	Adequate	82 (42.9)	109 (57.1)	191	

Values are given as n (%).

**Table 10:** Logistic regression for the relationship between adherence levels and demographic characteristics.

Variable (reference category)	*p*-value	AOR	95% CI for AOR	
			Lower	Upper
District *(Amuru)*				
Kampala	0.000	0.033	0.011	0.095
Kyotera	0.368	0.623	0.222	1.746
Wakiso	0.014	0.255	0.085	0.762
Knowledge level *(low)*				
High	0.000	27.599	5.12	148.784
Attitude level *(poor)*				
Good	0.010	2.56	1.252	5.236
Age (years) *(15–29)*				
30–44	0.677	0.849	0.393	1.834
45–59	0.993	0.995	0.343	2.883
>59	0.537	0.676	0.196	2.34

AOR, adjusted odds ratio; CI, confidence interval.

This study revealed that people who received COVID-19 news mainly from the media, such as the radio and television, were twice as likely to have adequate knowledge compared to those who relied on their friends for information. This might have been due to the fact that the trusted and reliable sources of information, i.e., the Ministry of Health and COVID-19 stakeholders, disseminated appropriate information mainly through media houses (27). Similarly, a study conducted in 2020 in Uganda reported a high knowledge score among people with phones connected to the Internet (28). In addition, this study revealed that the proportion of respondents with a high level of knowledge was higher among people with a formal education (who had attained at least a primary level), compared to those without a formal education. This was in agreement with the findings of Isah et al. in Nigeria, who reported that higher levels of education are associated with high knowledge of COVID-19 and its prevention guidelines (29).

The present study reported that most of the respondents strongly agreed that masks can help prevent COVID-19. This agreement with COVID-19 prevention measures, however, did not mean adherence to these measures as this study found that the level of adherence was low. These study findings were in agreement with the findings by Mboya et al., which revealed that public mask-wearing was regarded as an effective measure of stopping the spread of the virus when public compliance is high (25). Furthermore, a systematic review in Kenya found that the use of facemasks was very pertinent in preventing the spread of COVID-19 (30). That study also reported that the majority of people strongly agreed that they were at risk of getting COVID-19, and they were aware that the disease could be avoided mainly through hand washing, physical social distancing, as well as institutional quarantine.

### Study limitations

The community had already been in lockdown for months due to COVID-19 and hence was fragile. This resulted in low counts of respondents in some districts. Due to this situation, we also only considered the questions the respondents were comfortable responding to, causing fluctuations in the totals.

## Conclusion

The findings revealed multiple facilitators and barriers to adherence to PHSM. Factors such as good attitude and following media reports were considered to be promoters, while sufficient knowledge of COVID-19 among respondents facilitated adherence to PHSM. Respondents with low levels of knowledge of COVID-19 need to be targeted, to improve their attitude and adherence to safe practices. The platform to be used for communication on COVID-19 to the public should be radios, given that most respondents reported using them as their source of information.

## Data Availability

The original contributions presented in the study are included in the article/Supplementary Material. Further inquiries can be directed to the corresponding author.
